# Control Modification of Grasp Force Covaries Agency and Performance on Rigid and Compliant Surfaces

**DOI:** 10.3389/fbioe.2020.574006

**Published:** 2021-01-13

**Authors:** Raviraj Nataraj, Sean Sanford

**Affiliations:** ^1^Movement Control Rehabilitation Laboratory, Stevens Institute of Technology, Hoboken, NJ, United States; ^2^Department of Biomedical Engineering, Stevens Institute of Technology, Hoboken, NJ, United States

**Keywords:** cognitive agency, hand grasp force, movement rehabilitation, visual feedback, precision pinch

## Abstract

This study investigated how modifications in the display of a computer trace under user control of grasp forces can co-modulate agency (perception of control) and performance of grasp on rigid and compliant surfaces. We observed positive correlation (*p* < 0.01) between *implicit agency*, measured from time-interval estimation for intentional binding, and *grasp performance*, measured by force-tracking error, across varying control modes for each surface type. The implications of this work are design directives for cognition-centered device interfaces for rehabilitation of grasp after neurotraumas such as spinal cord and brain injuries while considering if grasp interaction is rigid or compliant. These device interfaces should increase user integration to virtual reality training and powered assistive devices such as exoskeletons and prostheses. The modifications in control modes for this study included changes in force magnitude, addition of mild noise, and a measure of automation. Significant differences (*p* < 0.001) were observed for each surface type across control modes with metrics for implicit agency, performance, and grasp control efficiency. Explicit agency, measured from user survey responses, did not exhibit significant variations in this study, suggesting implicit measures of agency are needed for identifying co-modulation with grasp performance. Grasp on the compliant surface resulted in greater dependence of performance on agency and increases in agency and performance with the addition of mild noise. Noise in conjunction with perceived freedom at a flexible surface may have amplified visual feedback responses. Introducing automation in control decreased agency and performance for both surfaces, suggesting the value in continuous user control of grasp. In conclusion, agency and performance of grasp can be co-modulated across varying modes of control, especially for compliant grasp actions. Future studies should consider reliable measures of implicit agency, including physiological recordings, to automatically adapt rehabilitation interfaces for better cognitive engagement and to accelerate functional outcomes.

## Introduction

The healthy hand is capable of exquisite grasp force control in manipulating objects during activities of daily living (Hubbard et al., 2009). Following neuromuscular traumas, such as spinal cord or brain injury, it is critical to rehabilitate grasp function for maintaining quality of life. Rehabilitation often involves physical therapy with repetitive task practice to reformulate neuromotor connections (Shepherd, 2001). Advanced physical therapy may employ engaging platforms such as virtual reality (VR) (Sveistrup, 2004) or robotics (Saleh et al., 2017). Powered assistive devices may also be employed to restore function as with powered exoskeletons (Lucas et al., 2004; Heo et al., 2012; Nataraj and van den Bogert, 2017) or neuroprostheses that activate sensorimotor pathways (Marasco et al., 2018; Schofield et al., 2019) of the hand. The primary objective with assistive or rehabilitative technologies is to enhance control of the hand and increase functional ability to perform manual tasks. Improved motor control may be enacted from training the person to move better independently or with the assistance of a powered device. Regardless of the rehabilitation approach, the person should be cognitively engaged and integrated with the therapeutic platform or the assistive device (Moore and Fletcher, 2012; Nataraj, 2017; Nataraj et al., 2020a,b,c). Improved perception of involvement and control of movement should better ensure continued participation and positive functional outcomes (Doyle, 2002; Behrman et al., 2005).

Despite intuitive relation between cognitive integration to movement and greater functional performance, this concept has not been systematically investigated nor incorporated in standard rehabilitation protocols. True innovation in neuromotor rehabilitation would include methods that optimize user-device movement abilities while increasing user cognition of movement. Systematically identifying agency, perception of control, and adapting device control accordingly may produce more effective, cognition-driven rehabilitation. Methods that leverage cognitive factors, such as agency, may accelerate functional gains and increase clinical retention of such methods and devices, which depends on user perception of utility (Childress, 1973; Phillips and Zhao, 1993; Hughes et al., 2014).

Sense of agency, or the perception of control, has been studied in experimental constructs that relate actions to consequences (Moore and Haggard, 2008; Moore, 2016). These studies have investigated modulation of agency with external cues (Moore et al., 2009; Khalighinejad et al., 2017) and the existence of agency within human machine interfaces (Evans et al., 2015; Le Goff et al., 2018). Agency is naturally implicated with rehabilitation through perception of neuromuscular action and related functional consequences (Moore and Obhi, 2012). Agency contributes to the performance of functional movements such as reaching (Nataraj et al., 2020c,d) and is impaired in the presence of neurological disorders (Jeannerod, 2009; Ritterband-Rosenbaum et al., 2012). Agency can also be compromised during the use of powered assistive devices, such as exoskeletons (Hartigan et al., 2015) or sensorimotor prostheses (Antfolk et al., 2013; Hebert et al., 2013), due to distortions in embodiment (Kilteni et al., 2012; Caspar et al., 2015). It remains unclear how agency is related to functional performance of grasping, and how agency and performance may be modulated with varying levels of control. Establishing the connection between agency and grasp could inspire the development of rehabilitation platforms that leverage agency for more effective control of grasp. These platforms would utilize agency to maximize classical performance objectives such as minimal effort or better movement tracking (Nataraj and van den Bogert, 2017).

Implicit measures of agency may be best utilized for adapting rehabilitation paradigms for grasp since they are less prone to conscious response bias (Wegner, 2003; Saito et al., 2015) compared to explicit measures of agency, which require survey-type responses (Moore et al., 2012). Indirect markers of agency, such as intentional binding, may better explain underlying feelings of control that are sensitive to sensory cues (Moore and Fletcher, 2012) and during impaired function as with neuropathological grasp (Delevoye-Turrell et al., 2002). Intentional binding indicates how coupled in time one perceives a voluntary action to an expected sensory consequence (Haggard et al., 2002; Moore and Obhi, 2012). Time-interval estimation between action and consequence has become a standard to implicitly infer agency via intentional binding. In the seminal work (Haggard et al., 2002), participants judged the time duration between an action (keypress) and sensory consequence (sound tone). A perceptual shift toward time compression was observed when the action was voluntary (high agency) versus an involuntary twitch (low agency) from transcranial magnetic stimulation. Intentional binding has since been used to explore agency in various contexts, including the influence of sensorimotor processes on agency from internal predictions and external outcomes (Moore and Haggard, 2008; Frith and Haggard, 2018).

Time-interval estimation methods for implicit measurements of agency are well posed for rehabilitation training. These methods can quantify agency trial-to-trial and are classically used with sensory feedback experiments. These experiments are similar to motor rehabilitation protocols employing external reward and sensory cues through VR (Sveistrup, 2004; Saleh et al., 2017). Any programmable interface for rehabilitation training or assistive device tuning can potentially adapt parameters for greater agency. Parameters include feedback gains (Nataraj et al., 2010, 2012b; De Havas et al., 2018) or customized settings within training environments (Velazquez et al., 2008). Systematic and computational approaches to adapt user training through agency would readily apply to any advanced rehabilitation platform (VR, robotics) or powered assistive devices such as exoskeletons (Farris et al., 2013) and neuroprostheses (Nataraj et al., 2012a,b; Marasco et al., 2018). The objective of agency-based rehabilitation would be to leverage perception of control for more effective user performance of functional tasks involving hand grasp. However, it remains unclear if varying control modes can effectively co-modulate agency and performance of grasp.

In this study, we hypothesized that agency was positively related to performance of a grasp force task. To test this hypothesis, we varied the control of a grasp force trace that participants visually tracked to match a target ramp. Error to the ramp served as the primary performance metric. We sought to observe potential covariation of agency and grasp force performance across various control modes. The testing environment utilized a force-sensitive pinch apparatus to record forces that were visually projected under the terms of each control mode. Completion of the ramp signified an “action” to be coupled to sensory “consequences” (visual and sound events) from which users estimated lapsed time intervals to implicitly infer agency via intentional binding. Each control mode defined the speed the force trace would move proportional to grasp force and if there existed a measure of noise or assisted automation. These control modes are consistent with parameters commonly adapted for powered devices such as setpoints for speed (Blaya and Herr, 2004; Wege et al., 2005), noise mitigation (Taylor et al., 2002; Agostini and Knaflitz, 2012), and automated assistance (Ronsse et al., 2011). These parameters can be tuned ad hoc (Terenzi, 1998) or identified through optimization of mechanical performance (e.g., effort, tracking) in a model system (Davoodi et al., 2007; Nataraj and van den Bogert, 2017). This study may newly inspire a cognitive basis from which to adapt such parameters in the rehabilitation of grasp performance.

The protocol in this grasp study was repeated for both a rigid and compliant surface. Compliance has been extensively considered for the object being grasped (Friedman and Flash, 2007; Nataraj et al., 2015) and in the design of robotic hands (Kazemi et al., 2012) that better mimic natural human grasp. As such, we sought to investigate how compliance may additionally affect the covariation of agency and performance across control modes. We hypothesized that compliant surfaces may induce higher agency due to the freedom to express more dexterous manipulation. Ultimately, we were able to observe how specific control modes may uniquely affect agency and performance of grasp against a rigid and compliant surface.

## Materials and Methods

**FIGURE 1:**
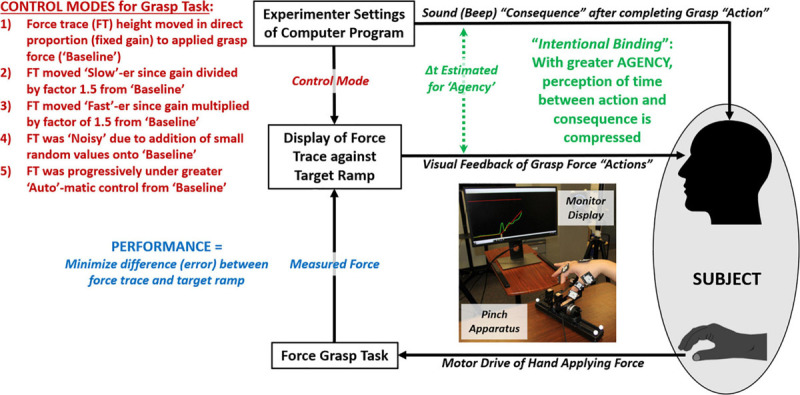
Flow diagram of experiment of participant performing grasp force task to visually trace a target ramp under varying control modes while assessing performance and agency.

The core experimental task involved participants controlling a visible trace to dynamically track a target ramp through precision pinch (index finger and thumb) grasp loading onto a force-sensitive pinch apparatus (Figure 1). Performance and agency were assessed across a variety of control modes for the force trace. An initial control mode, specified as “Baseline,” translated grasp loads to changes in trace height at a fixed gain. This gain was ∼2 vertical inches on the screen per 1 N total force applied. Total force was computed as the sum of the 3D (x-y-z) force vector of the index finger to that of the thumb, or:

$$\text{Total}\,\text{force}=\sqrt{f_{x,\text{index}}^{2}+f_{y,\text{index}}^{2}+f_{z,\text{index}}^{2}}+\sqrt{f_{x,\text{thumb}}^{2}+f_{y,\text{thumb}}^{2}+f_{z,\text{thumb}}^{2}}.$$

Other control modes were modifications from “Baseline” that involved changes in gain magnitude (and required peak force), addition of noise, or inclusion of automation. Participants were asked to maximize tracking performance in matching the force trace to a target ramp. Additionally, participants were required to apply a peak force that exceeded the top of the ramp to complete grasp “action” for each trial. This action would subsequently initiate a sound beep as “consequence” from which participants estimated the time-interval between action and consequence to assess agency. Based on intentional binding, the more subjects underestimate the time-interval, i.e., compress their perception of time, they exhibit greater agency in coupling their actions to related consequences.

### Participants

A total of 16 able-bodied volunteers (12 male, 4 female, 21 ± 3 years) were recruited to participate in this study. A power analysis for ANOVA at 95% suggested that seven-participant samples would show significant differences (α = 0.05) in agency and grasp force performance across the tested control modes. Only right-handed participants were tested for right-hand grasp to avoid effects of hand dominance. All participants had normal or corrected-to-normal vision and did not report nor demonstrate a history of disease, injury or complications involving cognition or upper extremity function. All participants signed an informed consent form for this study approved by the Stevens Institutional Review Board.

### Equipment (Hardware and Software)

**FIGURE 2:**
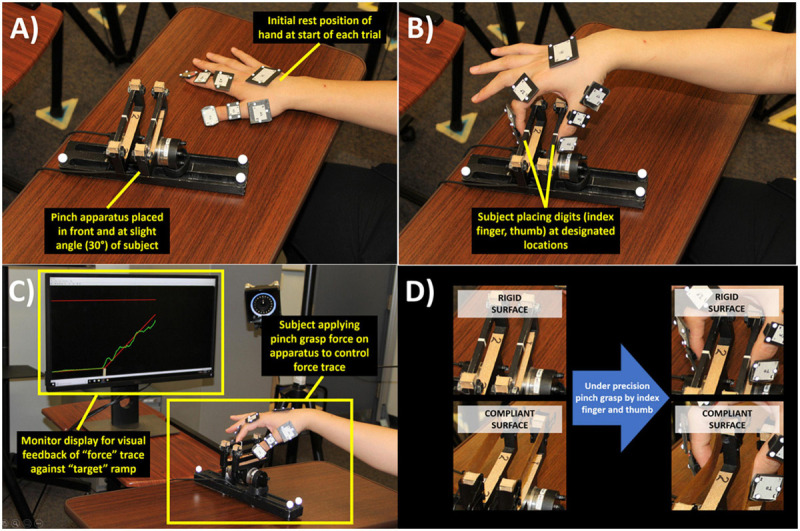
Participant applied grasp force to pinch apparatus each trial. **(A)** Participant hand initially palm-down on table to start each trial. **(B)** After trial began, participant moved hand to contact index finger and thumb on designated locations of grasp surfaces on pinch apparatus. **(C)** Participant progressively applied grasp force to control height (up-down) of force trace to match ramp as trial progressed. **(D)** Protocol repeated for rigid and compliant grasp surfaces.

A custom pinch apparatus (Figure 2) was constructed utilizing two 6-DOF load cells (Mini40, ATI Industrial Automation, Apex, NC, United States). The designated locations for applying pinch force included a surface for the index finger in parallel to a surface for the thumb. Both locations could accommodate surfaces as either a metal bar (rigid surface) or an elastic band (compliant surface). The band was set to provide approximately constant compliance of 1.5 N/cm normal to the surface. Data was acquired on a multi-input/output data acquisition system (PXIe-6363 with BNC interface, National Instruments, Austin, TX, United States). Force data was sampled at 100 Hz and processed in software developed in Simulink (Mathworks, Natick, MA, United States). The force trace was displayed in real-time on a 27-inch monitor (Dell P2717H).

### Protocol

#### Participant Preparation

After arriving to the laboratory, participants were re-informed about consent and had their right-hand size measured. Hand size was measured as the maximum spread distance from tip of thumb to tip of index finger. The average hand size was 15 ± 1 cm. For each participant, the distance between the index and thumb pinch surfaces was set at one-third of their hand size. Each participant was seated with chair height adjusted so the grasping hand could be table-supported with shoulders comfortably level. The pinch apparatus was kept in place on the table surface with double-side adhesive tape. The apparatus was positioned directly in front of the participant midline and oriented 30-degrees so that the index finger surface was comfortably forward and leftward to the thumb while grasping (Figure 2). The distance between the participant and the apparatus was set for a comfortable reach when grasping. The monitor displaying the force trace and ramp was placed approximately at participant eye level at a distance 1.5 m from the head.

#### Force Grasp Task

**FIGURE 3:**
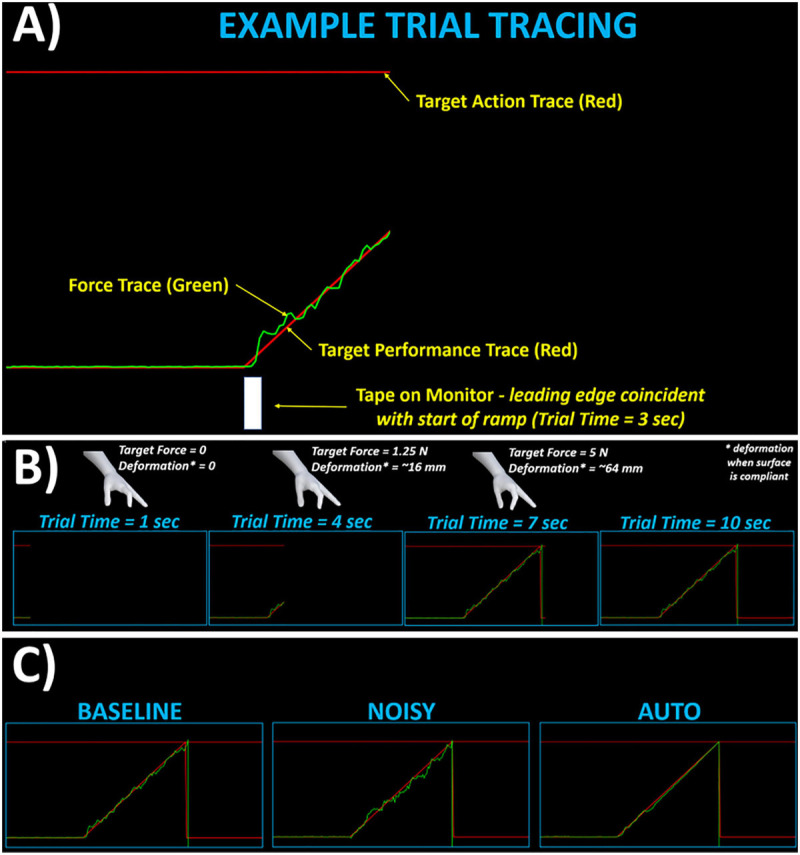
Snapshots of real-time tracings shown for various trial cases. **(A)** Example depiction of force and target (action, performance) traces shown during ramp portion of trial. **(B)** Tracing shown at various progressive instances across the 10-s trial. Also shown are depictions of respective grasp postures and force-deformation moduli assuming a “compliant” grasp surface. **(C)** Representative final traces shown for trials with “Baseline”, “Noisy”, and “Auto” control modes.

The experimenter cued the participant to the start of each trial, at which time, the participant would move the hand from rest, palm-side down on the table, to place their index finger and thumb near, without contact, designated locations on grasp surfaces of the apparatus. Each trial with data capture was 10 s. At the start of each trial (*t_*trial*_* = 0 s), the participant began to see real-time tracing of three lines. All three lines moved horizontally at a constant speed of 2.35 inches per second, computed as screen width divided by total trial time. The three lines (Figure 3) included: *(1) force trace (green line)* – the force trace height (upward vertical displacement) was under participant control and moved at fixed gain proportional to total grasp force. The force trace was additionally modified depending on the control mode applied. *(2) target performance trace (red line)* – this target trace was initially flat at height coincident with the force trace when no grasp forces were present. This target trace transitioned to a positive linear slope (ramp) from *t_*trial*_* = 3 s to *t_*trial*_* = 7 s. The ramp height grew at slope of ∼2 inches/sec over the 4-s ramp period. The bottom of the ramp coincided with zero force and the top with the target maximum force. *(3) target action trace (red line)* – this target trace was flat throughout the trial and remained at a height coincident with the target maximum force. This target maximum force was the same for all subjects since grasp forces were relatively low with maximum force around 5N. Variations in the maximum force were based on the specific control mode (described under “Varying control modes”). This target trace would meet the performance trace at the top of the ramp (*t_*trial*_* = 7 s).

The performance objective of the participant was to apply grasp forces to match, as best to their ability, the green force trace with the target performance trace. The participant was cued as to when the ramp would start by a piece of tape at the monitor base whose starting edge was coincident to *t_*trial*_* = 3 s. To successfully complete the trial and fulfill the agency objective, the participant needed to ensure the force trace crossed (contacted) the target action trace near (within 1 s) the top of the ramp. The crossing served as completion of the grasp “action” that then triggered a subsequent sound beep as “consequence” after some time-interval. The beep was moderately pitched with duration of 100 msec. The beep occurred at some time-interval between 100 and 1000 msec. The participant was asked to verbally estimate the time-interval to the best of their abilities after each beep. The participant was previously instructed that the time-interval was anywhere from 100 to 1000 msec in denominations of 100 msec. The actual time-intervals were always 100, 300, 500, 700, or 900 msec.

#### Varying Control Modes

All participants performed a block of trials of the grasp task for each of five different control modes, which were randomly presented. As previously described, the control modes examined in this study considered modifications in gain, addition of mild noise, and automation. The control modes were as follows:

(1) *Baseline* – The force trace magnitude (height) moved in direct proportion to the total grasp force applied at a gain of 2 in/N. The target maximum force associated with the top of the ramp at the end of 4-s period was 5 N. This control mode served as the template from which other control modes were modified.
(2) *Slow* – The force trace magnitude moved at a speed slower than “Baseline” for a given grasp load. Specifically, the gain was divided by 1.5 (reduced to 1.33 in/N) and the target maximum force consequently became 7.5 N. The participant needed to apply 50% more force on average than Baseline to accurately track the target ramp.
(3) *Fast* – The force trace magnitude moved at a speed faster than “Baseline” for a given grasp load. Specifically, the gain was multiplied by 1.5 (increased to 3 in/N) and the target maximum force consequently became 3.33 N. The participant needed to apply 33% less force on average than Baseline to accurately track the target ramp.
(4) *Noisy* – The force trace moved at the same speed as “Baseline” but was visually infected by mild noise. A small random value between (−0.5N, +0.5N) was added to each displayed instance of the force trace. This noise-level produced visible tremor that was noticeable but not distracting nor challenging in performing the grasp task.
(5) *Auto* – The force trace was progressively (linear with time) under greater automatic control. At the start-time of the ramp (*t*_*ramp*_ = 0, *t*_*trial*_ = 3 s), the participant controlled the force trace just as in “Baseline.” Over the 4-s ramp period, the force trace with “Auto” control (*FT*_*auto*_) was a weighted average between the participant’s force trace with “Baseline” control (*FT*_*base*_) and an optimal trace (*FT*_*opt*_) that perfectly matches the ramp. The displayed force trace for “Auto” was given as: $F\,T_{a\,u\,t\,o}=\left(1-\frac{t_{r\,a\,m\,p}}{4}\right)\times F\,T_{b\,a\,s\,e}+\left(\frac{t_{r\,a\,m\,p}}{4}\right)\times F\,T_{o\,p\,t}$. At *t*_*ramp*_ = 4 s, the force trace was guaranteed to match the top of the ramp and simultaneously match the target action line. This automated case was akin to user initiation of movement to trigger device assistance and auto-complete the movement (Farris et al., 2013).

#### Experimental Testing Blocks

Participants would perform a block of 20 consecutive trials for each of the five control modes. The first three trials of every block were “practice” with the time-interval between grasp action completion and the beep fixed at 1000 msec. The participant was aware these practice trials served to gain familiarity with the control mode and to re-calibrate their internal reference of a 1000 msec time-interval. The remaining 17 trials were used for agency and performance assessment with randomly presented time-intervals ranging from 100 to 900 msec with Gaussian distribution. Each participant was given up to 5 min between blocks to rest and complete a survey to rate their explicit subjective experience for the completed control mode. The trial-blocks for each of the control modes were conducted first for the rigid surface and then repeated for the compliant surface.

#### Surveys

For each trial-block, the participant was presented with a 1-statement survey to express their subjective perception of the control mode presented. Participants were asked to rate, on a 5-point Likert scale (−2 = strongly disagree, −1 = disagree, 0 = neutral, +1 = agree, +2 = strongly agree), to what extent the observed force trace movements reflected their intentions. The survey responses served as an explicit, or conscious, measure of agency (Moore et al., 2012) for comparison to the implicit measurements of agency.

### Data and Statistical Analysis

There were four data variables serving as the primary metrics in this study as follows:

(1) *Implicit agency (msec)* was the underestimation in time-interval between “action” (completion of force ramp) and “consequence” (delayed sound beep) to signify greater intentional binding. This measurement was taken once with each trial.
(2) *Performance (N^–1^)* was the inverse of the grasp force error during the 4-s ramp period to signify greater force tracking. Each measurement was taken as the mean error per trial.
(3) *Control efficiency (sec^2^/N^2^)* was the normalization of performance by force acceleration (N/sec^2^) to signify the error per unit acceleration effort to make corrections in tracking a constant velocity ramp. This variable was computed concurrently with performance.
(4) *Explicit agency (Likert scale)* was the survey response score on subjective perception of control mode. This measurement was done once after each block of trials.

The analyses that were performed on the above metrics are as follows:

*Analysis 1:* A linear regression was applied to performance (*y*-axis) and implicit agency (*x*-axis) data to assess the dependence of performance on agency for each surface in the *aggregate* (across all control modes, subjects). The *F*-statistic and *p*-value were computed to refute the null hypothesis that the slope coefficient was equal to zero and suggest significant dependence of performance on implicit agency. ANCOVA was performed to assess significant difference in slopes between compliant and rigid surfaces and to determine if significant slopes were observable within each control mode, not just in the aggregate.

*Analysis 2:* We performed a repeated-measures two-way ANOVA (factors for surface-type and control mode) for each metric to observe main effects due to each factor and potential interactions between factors. For significant factors, *post hoc* pairwise comparisons were done with Bonferonni correction for multiple comparisons. For multiple comparisons, all reported *p*-values are scaled according to the number of comparisons such that first-level significance is always *p* < 0.05. *Post hoc* comparisons allowed for observation of specific simple effects to be considered for each pair of control modes within surface type.

*Analysis 3:* Finally, the mean variability (standard deviation) in the force profile in each of the three directional dimensions was compared between rigid and compliant surfaces to indicate the presence of any surface-unique directional sensitivities during grasp.

## Results

### Analysis 1

**FIGURE 4:**
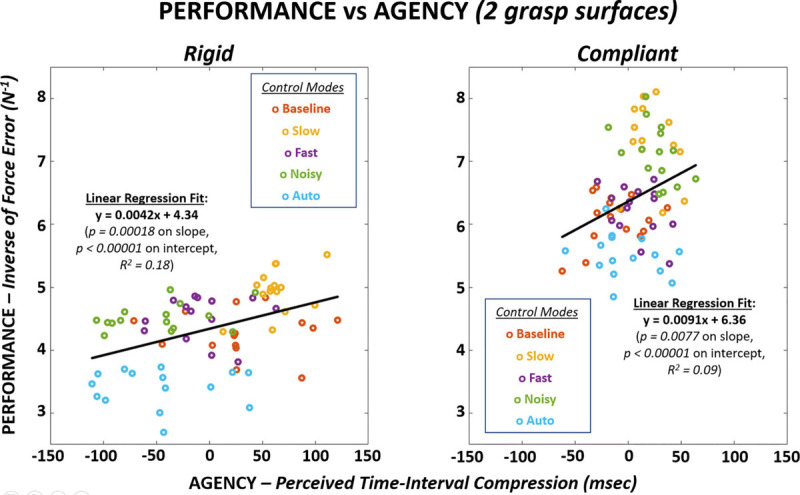
Performance of precision pinch grasp displayed against implicit agency across control modes on both *rigid* (LEFT) and *compliant* (RIGHT) grasp surfaces. Each point indicates a participant-average for that control mode and surface. Performance positively measured as inverse of average tracking error to target force ramp. Implicit agency positively measured as underestimation of time-intervals between completion of ramp task and subsequent sound beep.

The aggregate dependence of performance on agency across participant-averages for control modes is shown for each grasp surface in Figure 4. For both surfaces, there was a positive relationship between performance and agency indicated by a non-zero (*p* < 0.001) regression slope, however, the regression fit to both data sets was low (*R*^2^ < 0.20). Dependence of performance on agency was greater (increased slope) for the compliant surface. The increased slope with compliant surface grasp was confirmed with an ANCOVA comparison (Table 1B, *p* < 0.01). ANCOVA did not reveal significant slope dependence within control modes (Table 1A). ANCOVA did demonstrate significant differences in the intercept parameters both within control modes and in the aggregate across surfaces (Tables 1C,D). Combined slope and intercept results suggest that independent regressions for control modes are parallel but different, and that intercept differences across control modes drive differences in aggregate slope.

**TABLE 1A:** Linear regression results on slope (N^–1^ msec^–1^) for each control mode and ANCOVA results across control modes for each surface.

	**CONTROL MODE**					**ANCOVA**	
**SURFACE**	**Baseline**	**Slow**	**Fast**	**Noisy**	**Auto**	***F*-statistic**	***p*-Value**
Rigid	−3E−04	7E−03	3E−04	2E−03	−1E−04	0.55	0.457
Compliant	4E−03	6E−04	−7E−03	−1E−02	−3E−03	1.3	0.278

**TABLE 1B:** Linear regression results on slope (N^–1^ msec^–1^) in total (pooled) for each surface and ANCOVA results across both surfaces.

**SURFACE**		**ANCOVA**	
**Rigid**	**Compliant**	***F*-statistic**	***p*-Value**
0.0042	0.0091	3.04	**3E-03**

**TABLE 1C:** Linear regression results on intercept (N^–1^) for each control mode and ANCOVA results across control modes for each surface.

	**CONTROL MODE**					**ANCOVA**	
**SURFACE**	**Baseline**	**Slow**	**Fast**	**Noisy**	**Auto**	***F*-statistic**	***p*-Value**
Rigid	4.27	4.52	4.54	4.60	3.40	64.9	**7.7E-61**
Compliant	6.12	7.22	6.29	7.41	5.50	97.3	**3.8E-72**

**TABLE 1D:** Linear regression results on intercept (N^–1^) in total (pooled) for each surface and ANCOVA results across both surfaces.

**SURFACE**		**ANCOVA**	
**Rigid**	**Compliant**	***F*-statistic**	***p*-Value**
4.34	6.36	18.2	**2.3E-39**

*Significant *post hoc p*-values (<0.05) bolded and reported with Bonferonni correction.*

### Analysis 2

The two-way (factors of control mode, surface) ANOVA results for each of the primary metrics are shown in Table 2. A significant difference (*p* < 0.001) was observed with control mode for implicit agency, performance, and control efficiency. A significant difference (*p* < 0.001) was observed with surface for performance and control efficiency. In each case, the interaction term was significant and required an investigation of simple effects (i.e., hold one factor constant) and *post hoc* pairwise comparisons.

**TABLE 2:** Two-way ANOVA results for each metric over factors of control mode and surface.

	**METRIC**							
	**Implicit agency**		**Performance**		**Control efficiency**		**Explicit agency**	
**FACTOR**	***F*-statistic**	***p*-Value**	***F*-statistic**	***p*-Value**	***F*-statistic**	***p*-Value**	***F*-statistic**	***p*-Value**
Control mode	15.19	**1E-05**	76.8	**<1E-05**	16.4	**1E-05**	2.33	0.06
Surface	2.53	0.114	1054	**<1E-05**	2328	**1E-05**	∼0	∼1
Interaction	16.7	**1E-05**	6.7	**5E-05**	5.9	**2E-04**	0.73	0.57

*Significant *post hoc p*-values (<0.05) bolded and reported with Bonferonni correction.*

**FIGURE 5:**
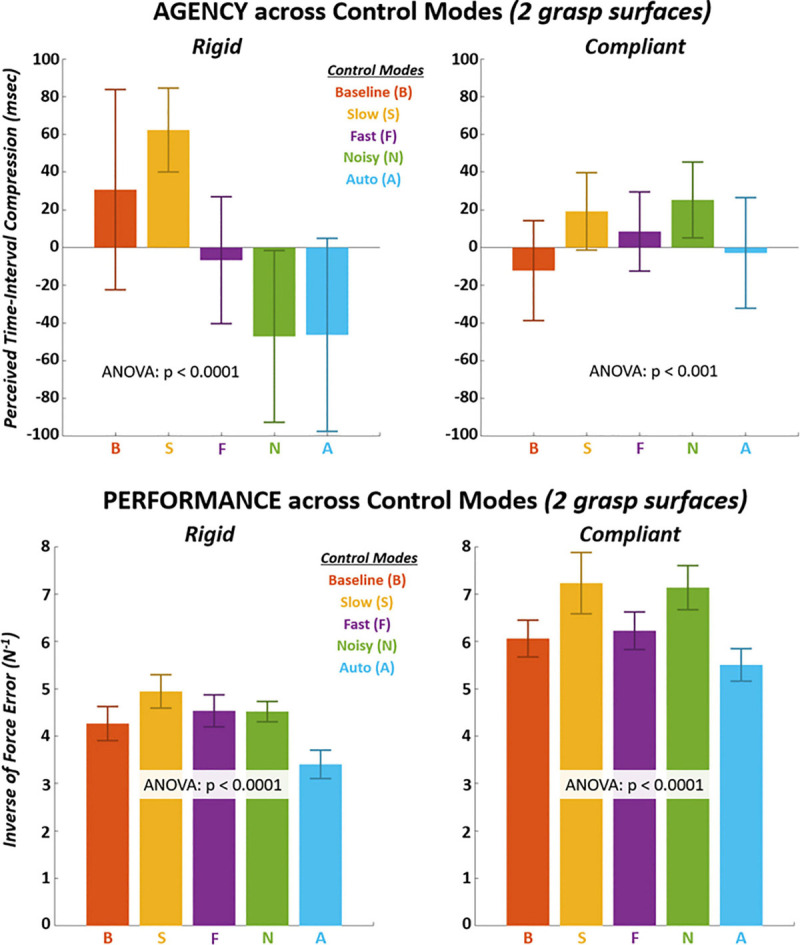
TOP – Mean implicit agency shown for each control mode on each grasp surface. Implicit agency positively measured as underestimation of time-intervals between completion of ramp task and subsequent sound beep. BOTTOM – Mean performance shown for each control mode on each grasp surface. Performance positively measured as inverse of average tracking error to target force ramp.

Several significant pairwise differences (*p* < 0.0001) were observed in *post hoc* across control modes for both implicit agency (Figure 5 TOP and Tables 3A–C) and performance (Figure 5 BOTTOM and Tables 3D–F). In the presence of significant interaction between surface and control mode, *unique* variations were observed across control modes based on surface for both implicit agency and performance. Performance was universally greater for the compliant surface than rigid surface. For the rigid surface, the highest agency and performance with significant pairwise differences (*p* < 0.0001) were observed for the “Slow” control mode. For the compliant surface, the highest agency and performance with significant pairwise differences (*p* < 0.001) were observed for the “Slow” and “Noisy” control modes.

**TABLE 3A:** Mean implicit agency (time-interval underestimation, *msec*) across control modes on rigid and compliant surfaces.

	**CONTROL MODE**					**ANOVA**		
**SURFACE**	**Baseline**	**Slow**	**Fast**	**Noisy**	**Auto**	***F*-statistic**	***p*-Value**	**η^2^**
Rigid	31 ± 53	62 ± 22	−7 ± 34	−47 ± 45	−46 ± 51	18.9	1.4E-10	0.52
Compliant	−12 ± 27	19 ± 21	8 ± 21	25 ± 20	−3 ± 29	6.3	2.2E-04	0.26

**TABLE 3B:** *Post hoc* comparisons (*p*-values) for implicit agency across control modes on rigid surface.

	**CONTROL MODE**				
**CONTROL MODE**	**Baseline**	**Slow**	**Fast**	**Noisy**	**Auto**
Baseline	–	0.26	0.13	**4E-05**	**5E-05**
Slow	–	–	**3E-04**	**2E-08**	**2E-08**
Fast	–	–	–	0.08	0.09
Noisy	–	–	–	–	0.99

**TABLE 3C:** *Post hoc* comparisons (*p*-values) for implicit agency across control modes on compliant surface.

	**CONTROL MODE**				
**CONTROL MODE**	**Baseline**	**Slow**	**Fast**	**Noisy**	**Auto**
Baseline	–	**5E-03**	0.13	**5E-04**	0.82
Slow	–	–	0.73	0.96	0.09
Fast	–	–	–	0.32	0.69
Noisy	–	–	–	–	**0.02**

**TABLE 3D:** Mean performance (inverse force error, N*^–^*^1^) across control modes on rigid and compliant surfaces.

	**CONTROL MODE**					**ANOVA**		
**SURFACE**	**Baseline**	**Slow**	**Fast**	**Noisy**	**Auto**	***F*-statistic**	***p*-Value**	**η^2^**
Rigid	4.3 ± 0.36	4.9 ± 0.35	4.5 ± 0.34	4.5 ± 0.22	3.4 ± 0.30	48.9	1.4E-19	0.73
Compliant	6.1 ± 0.39	7.2 ± 0.64	6.2 ± 0.40	7.1 ± 0.47	5.5 ± 0.34	38.4	5.7E-17	0.69

**TABLE 3E:** *Post hoc* comparisons (*p*-values) for performance across control modes on rigid surface.

	**CONTROL MODE**				
**CONTROL MODE**	**Baseline**	**Slow**	**Fast**	**Noisy**	**Auto**
Baseline	–	**1E-06**	0.15	0.20	**1E-08**
Slow	–	–	**6E-03**	**4E-03**	**1E-08**
Fast	–	–	–	0.99	**1E-08**
Noisy	–	–	–	–	**1E-08**

**TABLE 3F:** *Post hoc* comparisons (*p*-values) for performance across control modes on compliant surface.

	**CONTROL MODE**				
**CONTROL MODE**	**Baseline**	**Slow**	**Fast**	**Noisy**	**Auto**
Baseline	–	**2E-08**	0.86	**2E-07**	**1E-02**
Slow	–	–	**8E-07**	0.98	**1E-08**
Fast	–	–	–	**8E-06**	**5E-04**
Noisy	–	–	–	–	**1E-08**

*Significant *post hoc p*-values (<0.05) bolded and reported with Bonferonni correction.*

**FIGURE 6:**
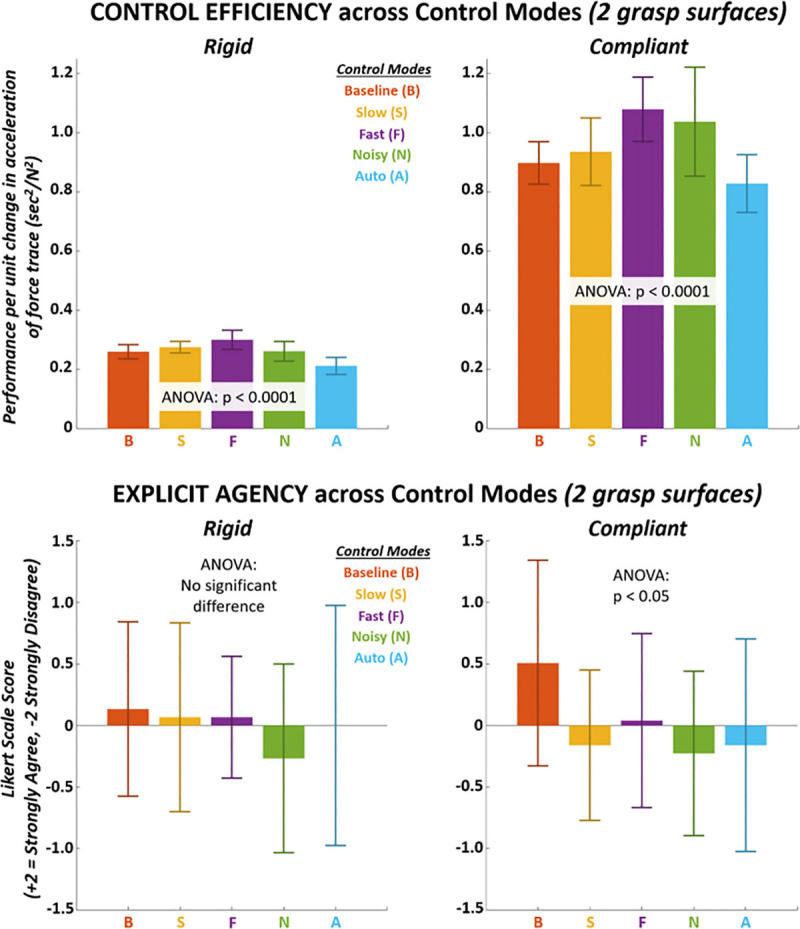
TOP – Mean control efficiency shown for each control mode on each grasp surface. Control efficiency measured as performance normalized by acceleration (2nd derivative) of force trace. BOTTOM – Mean explicit agency shown for each control mode on each grasp surface. Explicit agency measured from survey response along *Likert scale: +2 strongly agree, +1 agree, 0 neutral, –1, disagree, –2 strongly disagree*. Survey asked participant to what extent they agreed that force trace movements reflected their intended actions after a trial-block for each control mode.

Significant pairwise differences (*p* < 0.0001) were observed in control efficiency (Figure 6 TOP and Tables 4A–C) across control modes for both rigid and compliant surfaces. Similar to performance, there was significant interactions between surface and control mode for control efficiency such that unique variations in efficiency across control modes were observed for each surface. Furthermore, performance efficiency was also universally greater for the compliant surface. The lowest control efficiency was observed with “Auto” control mode for both surfaces and with multiple significant (*p* < 0.0001) pairwise differences. Significant differences (*p* < 0.05) were not observed for explicit agency (Figure 6 BOTTOM and Table 4D) except for the compliant surface which demonstrated one significant (*p* < 0.05) pairwise difference (“Baseline” greater than “Noisy”). As indicated from the two-way analysis, both surface effects and interaction with control modes were absent for explicit agency.

**TABLE 4A:** Mean control efficiency (*sec^2^/N^2^*) across control modes on rigid and compliant surfaces.

	**CONTROL MODE**					**ANOVA**		
**SURFACE**	**Baseline**	**Slow**	**Fast**	**Noisy**	**Auto**	***F*-statistic**	***p*-Value**	**η^2^**
Rigid	0.26 ± 0.02	0.28 ± 0.02	0.30 ± 0.03	0.26 ± 0.03	0.21 ± 0.03	19.6	**7E-11**	0.53
Compliant	0.90 ± 0.07	0.94 ± 0.11	1.1 ± 0.11	1.0 ± 0.18	0.83 ± 0.10	10.7	**8E-07**	0.38

**TABLE 4B:** *Post hoc* comparisons (*p*-values) for control efficiency across control modes on rigid surface.

	**CONTROL MODE**				
**CONTROL MODE**	**Baseline**	**Slow**	**Fast**	**Noisy**	**Auto**
Baseline	–	0.59	**1E-03**	0.99	**1E-04**
Slow	–	–	0.12	0.67	**4E-07**
Fast	–	–	–	**3E-03**	**1E-08**
Noisy	–	–	–	–	**8E-05**

**TABLE 4C:** *Post hoc* comparisons (*p*-values) for control efficiency across control modes on compliant surface.

	**CONTROL MODE**				
**CONTROL MODE**	**Baseline**	**Slow**	**Fast**	**Noisy**	**Auto**
Baseline	–	0.91	**1E-03**	**2E-02**	0.52
Slow	–	–	**2E-02**	0.16	0.12
Fast	–	–	–	0.88	**3E-06**
Noisy	–	–	–	–	**1E-04**

**TABLE 4D:** Mean explicit agency (*Likert scale: +2 strongly agree to −2 strongly disagree*) across control modes on rigid and compliant surfaces.

	**CONTROL MODE**					**ANOVA**		
**SURFACE**	**Baseline**	**Slow**	**Fast**	**Noisy**	**Auto**	***F*-statistic**	***p*-Value**	**η^2^**
Rigid	0.13 ± 0.71	0.07 ± 0.77	0.07 ± 0.49	−0.27 ± 0.77	0.0 ± 0.98	0.64	0.64	0.04
Compliant	0.51 ± 0.83	−0.16 ± 0.61	0.04 ± 0.71	−0.23 ± 0.67	−0.16 ± 0.86	2.5	**5E-02**	0.13

*Significant *post hoc p*-values (<0.05) bolded and reported with Bonferonni correction. Pairwise *post hoc* comparisons not reported for explicit agency **(D)** since neither main factor nor interaction demonstrated significant effect according to two-way ANOVA for this metric.*

**FIGURE 7:**
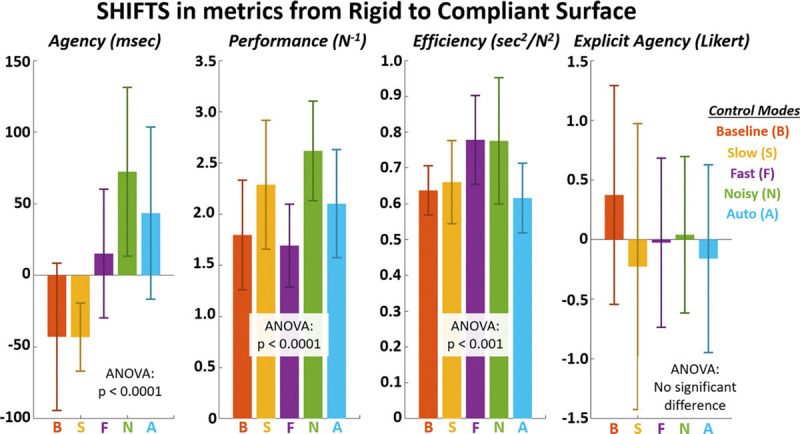
Shifts in metrics across control modes from rigid to compliant grasp surface. The surface-control mode *interaction p*-values are as follows: implicit agency = 1E-05, performance = 5E-05, efficiency = 2E-04, explicit agency = 0.57.

The shifts in metrics from rigid to compliant surfaces across control modes are explicitly shown in Figure 7 and Table 5. significant differences (*p* < 0.001) were observed for all metrics except for explicit agency (*p* > 0.05). The largest shifts for implicit agency, performance, and efficiency were observed for the “Noisy” control mode.

**TABLE 5:** Mean shifts from rigid to compliant surface for implicit agency (*msec*), performance (*N^–1^*), efficiency (*sec^2^/N^2^*), and explicit agency (*Likert*).

	**CONTROL MODE**				
**METRIC**	**Baseline**	**Slow**	**Fast**	**Noisy**	**Auto**
Implicit agency	−43 ± 51	−43 ± 23	15 ± 45	72 ± 59	43 ± 60
Performance	1.8 ± 0.53	2.3 ± 0.63	1.7 ± 0.41	2.6 ± 0.49	2.1 ± 0.52
Efficiency	0.63 ± 0.07	0.66 ± 0.12	0.78 ± 0.12	0.78 ± 0.18	0.6 ± 0.10
Explicit agency	0.37 ± 0.91	−0.23 ± 1.2	−0.03 ± 0.71	0.04 ± 0.66	−0.16 ± 0.79

### Analysis 3

**FIGURE 8:**
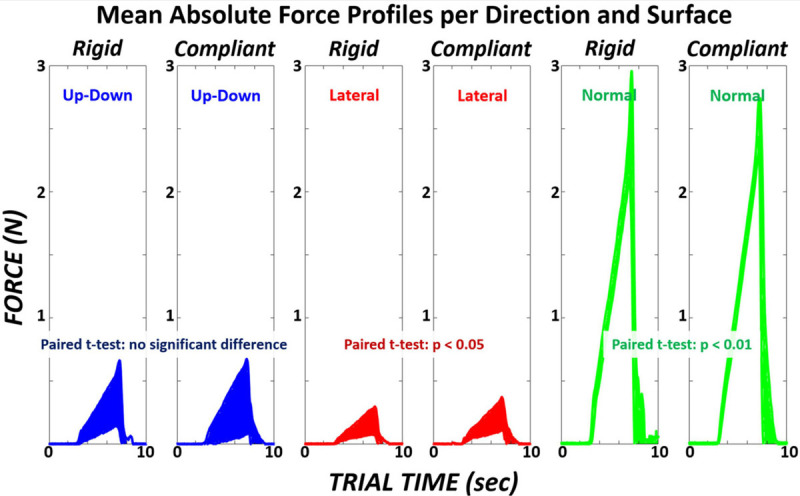
Mean absolute force profile across the 10-s trial for a single digit shown in each dimension (*up-down, lateral, normal*) for rigid versus compliant surfaces. Profile thickness indicates ±1standard deviation. Paired *t*-test performed across the average variability (standard deviation) for each dimension during ramp period (*t_*trial*_* = 3–7 s). The mean force variability in each dimension for the rigid surface was *0.294*, *0.098*, and *0.256 N*. The mean force variability in each dimension for the compliant surface was *0.291*, *0.142*, and *0.173 N*.

The mean absolute force profiles across a 10-s trial for a single digit are shown for each dimension and for rigid versus compliant surfaces in Figure 8. These mean force trajectories are taken across both digits and all subjects for the “Baseline” control mode. As expected, the greatest force was applied in the direction normal to the grasping surface. Significant differences (*p* < 0.05) in force variability (standard deviation) between surfaces were observed in the lateral and normal dimensions.

## Discussion

In this investigation of a precision grasp force task, we observed a positive relationship between implicit agency and performance and that both metrics can vary with modes of control. Furthermore, these general observations are consistent for grasp on either a rigid or compliant surface. While the positive dependence of performance on agency was significant and greater for the complaint surface, the regression fit was low. This suggests agency alone cannot “predict” performance and other explanatory variables are still needed. Furthermore, changes in control modes are required to elucidate this dependence, suggesting that agency can play a role in a dynamic framework for user-device adaptation. However, within a control mode whereby the user has presumably accommodated to a given condition (i.e., control mode), the performance-agency dependence may be diminished.

The dependence of performance on agency should motivate rehabilitation approaches that consider cognitive engagement beyond just entertainment and gamification (Sveistrup, 2004), but rather more efficient modes of physical therapy that restore neuromotor connectivity (Carter et al., 2012). Fostering motivation and engagement for greater participation is critical to ensure effective dosages of rehabilitation training (Hsieh et al., 2012; Stevenson et al., 2012). Our results additionally suggest that if cognition is systematically monitored and leveraged in real-time, the rehabilitation training sessions may further accelerate functional gains at a given level of participation.

Utilizing computerized interfaces, rehabilitation methods could readily leverage reliable, real-time cognitive measures with automated computational approaches. Optimization routines could be employed to systematically alter a VR training environment (Eddy and Lewis, 2002) with sensory feedback cues (visual, audio, haptic) that specifically enhance agency (Beck et al., 2017; Borhani et al., 2017; Wen et al., 2018). Presentation of avatars or goal-oriented tasks may be continually modified to elicit greater cognitive engagement while monitoring and promoting performance (Shin et al., 2014). Power-assistive exoskeletons and prosthetics driven by physiological commands (Lotte et al., 2012) may have input-output parameters [feedback gains, setpoint speeds, trajectories (Nataraj and van den Bogert, 2017)], customized to co-maximize agency and performance.

Virtual reality may be an appropriate platform to identify training settings and device parameters that maximize function and perception of control for rehabilitative and assistive interfaces (Sutcliffe and Kaur, 2000). Depending on the nature and extent of neuromuscular deficit, the training paradigm may be aimed toward either rehabilitating independent function or improving outcomes with a powered assistive device. In this study, VR training implications are specific to rehabilitation or powered assistance of hand grasp. Prevalent clinical populations include persons with impaired grasp due to hemiparesis, cervical-level spinal cord injury, or upper-limb amputation (Ma et al., 2014). In this study, we observed grasp movement training through a computerized interface with variations in control modes and surface types.

The factors of control modes and surface type demonstrated significant interaction in having significant effects on implicit agency, performance, and control efficiency. As such, it was necessary to observe simple effects with each factor while holding the other factor constant. Participants in this study demonstrated significantly greater hand grasp performance, efficiency of performance, and performance dependence on agency when grasping a compliant surface. Several investigations in hand grasp robotics employ algorithms that command actuation based on compliance (Cutkosky and Kao, 1989; Prattichizzo et al., 2012; Deimel and Brock, 2016). These approaches facilitate digit-level synergies that accommodate several degrees of freedom against a variety of grasp object sizes, shapes, hardness, and surface friction (Michelman and Allen, 1993; Kim et al., 2003; Dollar and Howe, 2006; Dollar et al., 2010). Our study suggests that compliance-based approaches are not only flexibly functional, but they may also encourage users to execute grasp with greater perception of control. While the shift in agency with compliant surfaces was negative for the “Baseline” and “Slow” modes of control, the largest singular shift was positive and observed with the “Noisy” mode.

Noise in sensory feedback can enhance functional performance (Priplata et al., 2002). However, visual noise is typically a distractor to impair performance (Vasilakos and Beuter, 1993; Baldassi et al., 2006). In our study, visual noise was relatively mild (magnitude <5% of target maximum force) and did not generate a significant change in performance from “Baseline” for the rigid surface. Visual noise did significantly reduce agency for the rigid surface, suggesting participants readily dissociated the visual noise from their own true actions (Miele et al., 2011). However, for the compliant surface, both agency and performance significantly increased from “Baseline.” The increase in agency suggests participants perceptually embodied the noise of the trace into the actions of their own digits (Caspar et al., 2015). Facilitating body representation with complex visual feedback can enhance movement performance (Sanford et al., 2020). Furthermore, the inability to dissociate the noise may be explained by the increased uncertainty introduced with the compliant surface. However, this erroneous perception of control did not reduce performance, but rather enhanced it. It is conceivable that while the noise did not represent true actions, the compliant surface may have amplified participant perception of freedom to enact greater control. In turn, this enhanced perception may have effectively increased performance responsivity, akin to increasing feedback gains on true visual error (Wei et al., 2005).

Unlike “Noisy,” the “Auto” control mode significantly reduced both performance and agency compared to “Baseline” across both surfaces. This finding suggests a sensitivity to gradual automation in grasp that reduced self-agency. The reduced perception of control appeared to reduce independent performance despite display of excellent performance with “Auto.” We posit that this performance reduction was not due to conscious awareness of automation since no significant differences in explicit agency were observed. The major implication for assistive devices is the importance of continuous user control in restoration of hand grasp. Traditionally, users generate a command beyond a threshold, with electromyography (EMG) (Marasco et al., 2018) or mechanical switches (Farris et al., 2013), to trigger a “go” command to the movement device (Geethanjali, 2016). The device will then automatically complete a movement sequence, such as grasp closure (Hart et al., 1998) or a step (Hartigan et al., 2015), without further user input until movement completion. While efficient in executing preprogrammed functions, interfaces with greater automation may severely hinder sense of user control and engagement to the device. More complex functions require nuanced user commands that may need to be identified through machine learning classification of EMG patterns (Zhou et al., 2017; Burns et al., 2019). However, even relatively simple tasks, such as ramped grasp force, may be benefited by more continuous user control to enhance agency and performance.

The “Slow” and “Fast” control modes effectively served as higher and lower grasp force ramps relative to “Baseline.” Changes in visual display were effectively uniform once participants accommodated to the load rate required to accurately track the ramp. Since control modes were randomly presented and required grasp loads were relatively small, we do not attribute metric differences for these modes due to learning or fatigue. Participants demonstrated significant increase in agency and performance with “Slow” compared to “Baseline” suggesting greater engagement and capability with higher grasp force. Previous studies have suggested greater agency may be facilitated through greater effort (Demanet et al., 2013). It is not clear if an optimal force level exists for precision pinch from this study. Our objective only sought to specify a low force pinch task and subsequently identify the changes in performance and agency due to control modifications that include changes in force level, addition of noise, and level of automation.

The major limitation of this study was examination of only one level for each control mode type. This study prioritized initial identification of agency and performance across a variety of mode types and two grasp surfaces. For fully customized deployment of an assistive device, it would be necessary to tune across multiple levels of each control mode concurrently. Control settings such as force magnitude, noise amplitude and frequency, and degree of automation may be specified independently or in unique combinations. The next phase of this research should establish methods that efficiently identify device settings such as optimal feedback gains with selective sampling (Nataraj et al., 2014). Optimal parameters could be determined to not only minimize performance errors for better movement (Neptune, 1999; Xiang et al., 2010). But to maximize quantifiable metrics for cognition. Our study suggests implicit agency may be such as agency for greater user-device integration during rehabilitation practices. An assumption of this study was that gender does not affect grasp performance or agency. Gender-based differences for movement agency and precision grasp have not been well established, but not considering gender as a factor is a potential study limitation.

Future studies should also consider alternative measurements of agency. While explicit metrics for agency have been commonly utilized in other studies (Dewey and Knoblich, 2014), variability was too large to identify significant differences in this study. This study did demonstrate that an implicit measure of agency, time-interval estimation for intentional binding, had correlation to performance in the aggregate and could be modulated across control modes. It remains unclear if this metric has sufficient sensitivity and resolution for customization of rehabilitation programs and devices to individual users. Furthermore, requiring each user to provide a verbal estimate of time-intervals would be tedious and cognitively fatiguing while adapting the training interface. The user should be able to devote greater attention to operating the interface while implicit agency is passively measured.

Physiological measures such as EMG and brain electroencephalography (EEG) may be monitored to reflect changes in cognitive agency (Tsakiris et al., 2006; Kang et al., 2015; Beyer et al., 2017). These measures need to be validated as a reliable surrogate for implicit agency and device-based rehabilitation interfaces. Computational optimization tools may be employed to concurrently adapt the user-device interface based on dynamic features of real-time EEG and EMG to assess cognitive integration. To this end, probabilistic methods that evaluate embodied cognition (Ryu and Torres, 2018) may be especially appropriate to extract perceptual features from stochastic physiological signals such as EEG and EMG. Reliable and validated physiological measures for agency would facilitate autonomous adaptation of rehabilitation interfaces for greater cognition. Interfaces include VR-based training paradigms and powered assistive devices such as prosthetics and exoskeletons.

## Data Availability Statement

The underlying data supporting the conclusions of this article will be made available by the authors, without undue reservation.

## Ethics Statement

The studies involving human participants were reviewed and approved by the Stevens Institutional Review Board. The patients/participants provided their written informed consent to participate in this study.

## Author Contributions

RN contributed to designing and developing the experiment, analyzing the data, writing and revising the manuscript, and directing the project. SS contributed to recruiting participants, performing the data collections, and revising the manuscript. Both authors contributed to the article and approved the submitted version.

## Conflict of Interest

The authors declare that the research was conducted in the absence of any commercial or financial relationships that could be construed as a potential conflict of interest.
